# Association between Adverse Childhood Experiences and long-term outcomes in people at Clinical High-Risk for Psychosis

**DOI:** 10.1038/s41537-025-00562-9

**Published:** 2025-02-20

**Authors:** Stefania Tognin, Ana Catalan, Claudia Aymerich, Anja Richter, Matthew J. Kempton, Gemma Modinos, Ryan Hammoud, Iñigo Gorostiza, Evangelos Vassos, Mark van der Gaag, Lieuwe de Haan, Barnaby Nelson, Anita Riecher-Rössler, Rodrigo Bressan, Neus Barrantes-Vidal, Marie-Odile Krebs, Merete Nordentoft, Stephan Ruhrmann, Gabriele Sachs, Bart P. F. Rutten, The EU-GEI High Risk Study, Stefania Tognin, Philip McGuire, Lucia R. Valmaggia, Matthew J. Kempton, Maria Calem, Gemma Modinos, Lieuwe de Haan, Mark van der Gaag, Eva Velthorst, Tamar C. Kraan, Daniella S. van Dam, Nadine Burger, Barnaby Nelson, Patrick McGorry, G. Paul Amminger, Christos Pantelis, Athena Politis, Joanne Goodall, Anita Riecher-Rössler, Stefan Borgwardt, Erich Studerus, Rodrigo Bressan, Ary Gadelha, Elisa Brietzke, Graccielle Asevedo, Elson Asevedo, Andre Zugman, Neus Barrantes-Vidal, Tecelli Domínguez-Martínez, Anna Racciopi, Thomas R. Kwapil, Manel Monsonet, Lídia Hinojosa, Mathilde Kazes, Claire Daban, Julie Bourgin, Olivier Gay, Célia Mam-Lam-Fook, Marie-Odile Krebs, Dorte Nordholm, Lasse Randers, Kristine Krakauer, Louise Glenthøj, Birte Glenthøj, Merete Nordentoft, Stephan Ruhrmann, Dominika Gebhard, Julia Arnhold, Joachim Klosterkötter, Gabriele Sachs, Iris Lasser, Bernadette Winklbaur, Philippe A. Delespaul, Bart P. Rutten, Jim van Os, Lucia Valmaggia, Philip McGuire

**Affiliations:** 1 Department of Psychosis Studies, Institute of Psychiatry, Psychology & Neuroscience, King’s College London, London, UK; 2 Basurto University Hospital, OSI Bilbao-Basurto. Biobizkaia Health Research Institute. University of the Basque Country UPV/EHU. Centro de Investigación en Red de Salud Mental (CIBERSAM), Instituto de Salud Carlos III, Barakaldo, Bizkaia Spain; 3 Department of Children & Adolescent Psychiatry, Institute of Psychiatry, Psychology & Neuroscience, King’s College London, London, UK; 4 National Institute for Health Research (NIHR) Biomedical Research Centre (BRC), London, UK; 5 Department of Psychological Medicine, Institute of Psychiatry, Psychology & Neuroscience, King’s College London, London, UK; 6 MRC Centre for Neurodevelopmental Disorders, King’s College London, London, UK; 7 Research Unit, Basurto University Hospital, REDISSEC, Bilbao, Spain; 8 Social, Genetic & Developmental Psychiatry Centre, Institute of Psychiatry, Psychology & Neuroscience, King’s College London, London, UK; 9 VU University, Faculty of Behavioural and Movement Sciences, Department of Clinical Psychology and Parnassia Psychiatric Institute, Department of Psychosis Research, Amsterdam, The Netherlands; 10 AMC, Academic Psychiatric Centre, Department Early Psychosis, Amsterdam, The Netherlands; 11 Orygen, Parkville, VIC Australia; 12 Centre for Youth Mental Health, The University of Melbourne, Parkville, VIC Australia; 13 Medical Faculty, University of Basel, Basel, Switzerland; 14 LiNC—Lab Interdisciplinar Neurociências Clínicas, Depto Psiquiatria, Escola Paulista de Medicina, Universidade Federal de São Paulo — UNIFESP, São Paulo, Brazil; 15 Departament de Psicologia Clínica i de la Salut (Universitat Autònoma de Barcelona), Spanish Mental Health Research Network (CIBERSAM), Barcelona, Spain; 16 University of Paris, GHU-Paris, Sainte-Anne, C’JAAD, Hospitalo-Universitaire department SHU, Paris, France; 17 Mental Health Centre Copenhagen and CINS, Mental Health Centre Glostrup, Mental Health Services in the Capital Region of Copenhagen, University of Copenhagen, Copenhagen, Denmark; 18 Department of Psychiatry and Psychotherapy, Faculty of Medicine and University Hospital, University of Cologne, Cologne, Germany; 19 Department of Psychiatry and Psychotherapy, Medical University of Vienna, Vienna, Austria; 20 Department of Psychiatry and Neuropsychology, School for Mental Health and Neuroscience, Maastricht University Medical Centre, Maastricht, The Netherlands; 21 Department of Psychology, Institute of Psychiatry, Psychology & Neuroscience, King’s College London, London, UK; 22 Department of Psychiatry, University of Oxford, Oxford, United Kingdom; 23 Parnassia Psychiatric Institute, Department of Psychosis Research, The Hague, The Netherlands; 24 Icahn School of Medicine at Mount Sinai, Department of Psychiatry, New York, NY 10029 USA; 25 Melbourne Neuropsychiatry Centre, University of Melbourne & Melbourne Health, Melbourne, VIC Australia; 26 Depto Psiquiatria, Escola Paulista de Medicina, Universidade Federal de São Paulo – UNIFESP, São Paulo, Brazil; 27 CONACYT-Dirección de Investigaciones Epidemiológicas y Psicosociales, Instituto Nacional de Psiquiatría Ramón de la Fuente Muñiz, México City, México; 28 Departament de Psicologia Clínica i de la Salut (Universitat Autònoma de Barcelona), Barcelona, Spain; 29 Department of Psychology, University of Illinois at Urbana-Champaign, Champaign, IL USA; 30 Centre for Neuropsychiatric Schizophrenia Research (CNSR) & Centre for Clinical Intervention and Neuropsychiatric Schizophrenia Research (CINS), Mental Health Centre Glostrup, University of Copenhagen, Glostrup, Denmark; 31 Psyberlin, Berlin, Germany; 32 Mondriaan Mental Health Trust, Heerlen, The Netherlands

**Keywords:** Psychosis, Schizophrenia

## Abstract

Adverse childhood experiences (ACEs) are common in people at clinical high-risk for psychosis (CHR), however, the relationship between ACEs and long-term clinical outcomes is still unclear. This study examined associations between ACEs and clinical outcomes in CHR individuals. 344 CHR individuals and 67 healthy controls (HC) were assessed using the Childhood Trauma Questionnaire (CTQ), the Bullying Questionnaire and the Childhood Experience of Care and Abuse (CECA). CHR were followed up for up to 5 years. Remission from the CHR state, transition to psychosis (both defined with the Comprehensive Assessment of an At Risk Mental State), and level of functioning (assessed with the Global Assessment of Functioning) were assessed. Stepwise and multilevel logistic regression models were used to investigate the relationship between ACEs and outcomes. ACEs were significantly more prevalent in CHR individuals than in HC. Within the CHR cohort, physical abuse was associated with a reduced likelihood of remission (OR = 3.64, *p* = 0.025). Separation from a parent was linked to an increased likelihood of both remission (OR = 0.32, *p* = 0.011) and higher level of functioning (OR = 1.77, *p* = 0.040). Death of a parent (OR = 1.87, *p* = 0.037) was associated with an increased risk of transitioning to psychosis. Physical abuse and death of a parent are related to adverse long-term outcomes in CHR. The counter-intuitive association between separation from a parent and outcomes may reflect the removal of a child from an adverse environment. Future studies should investigate whether interventions targeting the effect of specific ACEs might help to improve outcomes in this population.

## Introduction

Some of the most studied environmental and psychological risk factors for psychosis are Adverse Childhood Experiences (ACEs)^1^. ACEs include different traumatic experiences such as psychological and physical abuse and neglect, sexual abuse, parental loss or separation, and bullying. Several studies have consistently pointed to a heightened risk of psychosis associated with ACEs^2–10^, and that ACEs are related to negative outcomes in schizophrenia spectrum disorders, such as poor functioning and persistence of symptoms^11^. Mall and colleagues^12^ reported that individuals who experienced ACEs showed 2.44 times increased odds of developing schizophrenia compared to those who did not. These compelling findings not only suggest a plausible causal connection between ACEs and the onset of schizophrenia spectrum disorders but also highlight the critical need for in-depth exploration of the links between specific ACEs and their cumulative effects on psychosis outcomes.

Most previous studies on the role of Adverse Childhood Experiences (ACEs) on psychopathological long-term outcomes in psychotic disorders have relied on samples of patients with established psychosis. The extent to which these measurements are confounded by effects of illness duration and treatment, or by recall bias, is therefore unclear^13^. One way to reduce the potential effects of these confounders is to evaluate ACEs before the onset of illness, in people at clinical high-risk for psychosis (CHR).

ACEs are more common in CHR individuals than in healthy volunteers^14,15^, with a meta-analytical mean prevalence rate of 86.8%^16^. Furthermore, around 5% of this population also meets criteria for a comorbid post-traumatic stress disorder (PTSD)^17^. This is particularly relevant considering the increasing evidence suggesting that trauma exposure is associated to both transition to psychosis^18,19^ and higher severity of attenuated psychotic symptoms^20^. However, people at CHR may experience other adverse clinical outcomes, such as non-remission of symptoms and poor functioning at the 24-months follow-up^8,21,22^. Moreover, a larger proportion of the CHR population experience these outcomes than the minority that develop a psychotic disorder^21–23^. Childhood trauma has been consistently associated with worse functioning at an adult age, including poorer cognitive performance^24^, worse social functioning^25^ and health outcomes^26^, for both general and clinical populations. However, factors linked to non-remission or poor functioning have been much less studied than factors associated with increased risk of transitioning to psychosis.

In particular, social functioning and subjective quality of life are recognized as important treatment outcomes in psychosis and psychosis risk^27–29^. They have been described as global constructs or as individual’s ability to adapt to societal, familial, and professional demands. ACEs in individuals with psychosis have been associated with disruptions to social and academic functioning in adult life^30^, however, little is known about the contribution of ACEs to impaired social functioning in people at CHR of psychosis. Understanding the factors that contribute to and are associated with the emergence of negative medium- and long-term outcomes is critical for eventually developing interventions to prevent ACEs and developing psychological treatments designed to reduce the detrimental effects of ACEs.

The aims of the present study were to assess whether specific ACESs are related to adverse outcomes (non-remission of CHR symptoms, levels of social and occupational functioning, and transition to psychosis) in people at CHR. We tested the hypothesis that ACEs would be associated with non-remission of symptoms, low level of social and occupational functioning as well as with transition to psychosis.

## Methods

### Study design and participants

This was a multicentre, prospective study. ACEs and clinical characteristics were collected from participants in the The EUropean Network of National Schizophrenia Networks Studying Gene-Environment Interactions (EU-GEI) High Risk study^31^, a naturalistic prospective case-control study involving 11 sites in 6 countries (London, Amsterdam, The Hague, Basel, Cologne, Copenhagen, Paris, Barcelona, Vienna, Sao Paulo, and Melbourne). The sites were chosen to represent a blend of rural and urban regions, featuring diverse proportions of minority ethnic groups, and assessment measures were standardized across the different countries^32^. The total EU-GEI sample comprised 344 CHR individuals and 67 healthy controls (HC). For individuals at CHR, exclusion criteria were past or present diagnosis of psychotic disorders or neurological disorders and estimated intelligence quotient (IQ) lower than 60. For HC, inclusion criteria were not meeting criteria for the CHR state, no past or present diagnosis of psychotic disorders or neurological disorders and being recruited from the same geographical areas as the CHR group. Ethical approval was obtained from each site’s local research ethics committee. The study was conducted in accordance with the Declaration of Helsinki. All participants provided written informed consent.

### Clinical assessment and outcomes

At baseline, the Comprehensive Assessment of At-Risk Mental States (CAARMS)^33^ was used to determine whether participants met inclusion criteria for the CHR state. Participants meeting CHR criteria who were receiving antipsychotic medication were included, provided the medication had not been prescribed for a psychotic episode. Participants were also evaluated with the GAF disability scale^34^. Raters were trained in the use of the CAARMS and Global Assessment of Functioning (GAF) prior to the study and completed online training videos every 12 months from study onset to assess interrater reliability (see previous publication^35^). Data on age, sex, and race were obtained using the Medical Research Council Sociodemographic Schedule^36^. IQ was estimated using the shortened version of the Wechsler Adult Intelligence Scale^37^. Current and lifetime mental disorders were assessed using the Structured Clinical Interview for DSM-IV Axis I and II Disorders (SCID-I, SCID-II)^38,39^.

Participants were monitored for up to 5 years, with face-to-face interviews at 6, 12 and 24 months. Transition to psychosis was defined according to the CAARMS criteria^33^. Where possible, participants were assessed with the SCID-I at each planned follow-up to establish a formal diagnosis according to DSM-IV criteria^40^. This allowed to ascertain diagnosis of psychotic disorder or other psychiatric disorders throughout the duration of the study. If a participant was unable to attend a follow-up or was lost at follow-up, Electronical Health Care Records (eHCR) were used to determine if transition to psychosis had occurred. This information was identified in eHCR as a code (i.e. International Classification of Diseases code) or as a single entry (i.e. name of diagnosis). Remission from the CHR state was defined as an individual no longer meeting CAARMS inclusion criteria at the last available follow-up^41^. Level of functioning was defined using the GAF disability scale based on the last available follow-up data. GAF data were subsequently dichotomized into high and low GAF, with high GAF score equal or greater than 60 entailing better functioning in CHR samples^42,43^. This approach is in line with previous studies and reflect the clinical significance of the GAF scores (GAF $\ge$ 60 corresponds to relatively good functioning), ^44^.

### Adverse Childhood experiences (ACEs)

ACEs were assessed retrospectively using the Brief version of the Childhood Trauma Questionnaire (CTQ)^45^, the Childhood Experience of Care and Abuse (CECA-Q)^46^ and the Retrospective Bullying Questionnaire^47^.

The CTQ is a 25-item self-report questionnaire which assesses traumatic experiences before the age of 17. Individuals rated their level of exposure on a scale from 1 (never) to 5 (very often). This generated a total score, as well as subscores for five domains: emotional abuse (EA), physical abuse (PA), sexual abuse (SA), emotional neglect (EN) and physical neglect (PN). Cut-off scores were then used to classify individuals into groups based on the presence or absence of clinically significant histories of abuse and neglect. These moderate to severe thresholds have been previously used identify these cases while minimizing the risk of false positives^48,49^. Moderate-severe cut-off scores for each subscale were $\ge$ 13 for EA; $\ge$ 10 for PA; $\ge$ 8 for SA; $\ge$ 15 for EN; and >= 10 for PN^50^. Being identified as positive for a category corresponds with endorsing a substantive number of experiences as “often true”.

The CECA-Q assesses traumatic experiences such as the death of a parent, separation from parents (including being in foster care), parental discordance, lack of adult support, poverty, cruelty, and violence. These different measures of ACEs were categorized as present or absent.

The Retrospective Bullying Questionnaire measures the severity of the bullying experience (emotional, psychological, or physical violence) on a 0 to 3 score. Then, exposure to childhood bullying was dichotomized using $\ge$ as the cut-off point (0 = “absent” and $\ge$ 1 = present).

### Statistical analysis

All statistical analyses were performed using STATA version 17 (StataCorp, 2023). Demographic and clinical data were compared using independent *t*-tests for continuous variables and chi-square tests for categorical variables. Significant effects are reported at *p* < 0.05. To assess collinearity among the ACEs variables, we used the Variance Inflation Factor (VIF). Only ACEs items that did not show multicollinearity, as indicated by VIF values below 10, were included in the analyses.

An a priori sample size calculation was conducted based on a 95% confidence level, 5% margin of error, and 80% statistical power. Each outcome was dichotomous, and the approximate prevalence of each outcome was estimated from previously reported data in the literature. For the transition to psychosis in CHR populations, a prevalence of 19.0% was used^51^. For symptom remission in CHR, a prevalence of 33.4% was applied^52^. Lastly, a prevalence of 25.0% was chosen for poor functional prognosis^53^. This yielded required sample sizes of 237, 342, and 289 for each outcome, respectively.

We used the whole CHR sample to analyze the relationship between ACEs and transition to psychosis. Analyses of other outcomes were limited to participants for whom data on symptom remission and functioning were available. Specifically, the sample used to analyze the association between symptom remission and ACEs included 241 CHR individuals, 74.27% of whom completed a face-to-face assessment at 12 months, and 34.63% of those completed an additional assessment at 24 months. At the last follow-up, 70 showed symptom remission, and 171 still met CHR criteria. Healthy controls (HC) were not included in the transition and remission analyses. The sample available to analyze the association between functioning (measured using the Global Assessment of Functioning, GAF disability) and ACEs comprised 221 CHR individuals and 50 HC. Among the CHR, 68.33% were assessed face-to-face at 12 months, and 39.73% of those completed an additional assessment at 24 months. 122 CHR participants presented poor functioning and 99 good functioning while all HC presented good functioning. Participants without information on symptom remission or functioning were excluded from these analyses. In each analysis, sex, age, race and site were included as covariates. Approximate IQ was also included as a covariate, as evidence suggests that neurocognitive function is one of the primary determinants of functional outcome in CHR individuals^54^.

All outcomes were analysed using stepwise regression and multilevel logistic regression models with data clustered by site as a random intercept, to consider that observations from the same site could be correlated. In the first step we conducted a stepwise regression, including all the variables related to ACEs measures and socio-demographic variables that could potentially influence the outcomes (Table 1), which is a method of fitting regression models in which the choice of predictive variables is carried out by an automatic procedure. We used a significance level of 0.2^55^ for variables to enter and stay in the model. In the second step we performed a multilevel logistic regression (melogit) to analyze the binary outcomes based on a subset of variables from the stepwise regression and accounting for clustering by site. This approach allows for the examination of how specific factors are associated with the outcomes, controlling for the non-independence of observations within sites, and adjusting for potential confounding variables identified in the stepwise regression. Variables with a final significance *p* < 0.05 were considered significant in the model.

**Table 1:** Demographic and clinical characteristics of included participants.

	HC (*n* = 67)	CHR (*n* = 344)
Sex, *N* (%)		
Male (%)	34 (50.75)	185 (53.78)
Female (%)	33 (49.25)	159 (46.22)
Age in years, mean (SD)	23.00 (4.00)	22.00 (5.00)
Education in years, mean (SD)***	16.00 (3.00)	14 (3.00)
IQ (WAIS), mean (SD)***	113 (18.00)	98 (17.00)
Bullied frequently and severely yes, *N* (%)**	12 (17.91)	124 (38.87)
Physical Abuse yes, *N* (%)*	2 (3.03)	42 (13.21)
CTQ Physical Abuse Score, mean (SD)*	5.89 (2.16)	7.25 (3.61)
Physical Neglect yes, *N* (%)*	5 (7.58)	66 (20.75)
CTQ Physical Neglect Score, mean (SD)*	6.39 (2.47)	8.17 (3.14)
Emotional Abuse yes, *N* (%)***	5 (7.58)	114 (35.74)
CTQ Emotional Abuse Score, mean (SD)**	7.45 (3.12)	11.93 (5.20)
Emotional Neglect yes, *N* (%)**	8 (12.12)	100 (31.35)
CTQ Emotional Neglect Score, mean (SD)**	9.42 (4.72)	13.20 (4.81)
Sexual Abuse yes, *N* (%)**	4 (6.06)	65 (20.38)
CTQ Sexual Abuse Score, mean (SD)*	5.50 (1.92)	7.10 (4.23)
CTQ Total Score, mean (SD)***	34.67 (10.65)	47.66 (15.49)
Death of a parent yes, *N* (%)	4 (4.55)	25 (7.86)
Separation from a parent yes, *N* (%)	22 (33.33)	131 (40.81)
Taken into care yes, *N* (%)	2 (2.99)	16 (5.02)
Parental discord yes, *N* (%)*	29 (43.28)	185 (58.18)
Lack of adult support yes, *N* (%)***	14 (20.90)	143 (45.11)
Poverty yes, *N* (%)*	15 (22.39)	122 (35.44)

*CHR* clinical high-risk, *CHR-NT* clinical high-risk non-transition, *CHR-T* clinical high- risk transition, *CTQ* childhood trauma questionnaire, *HC* healthy controls, *SD* standard deviation.

*p < 0.05; **p < 0.01; ***p < 0.001.

## Results

### Demographic and clinical data

Socio-demographic characteristics of the sample are detailed in Table 1. HC had more years in education, higher IQ, and higher GAF scores than CHR individuals. Most ACEs were significantly more frequent in the CHR group, except for death of a parent, separation from a parent, and being taken into care. 65 individuals transitioned to psychosis during the follow-up (CHR-T) and 279 did not (CHR-NT). The mean time to transition to psychosis was 373.14 days (SD = 404.10). Black race was associated with increased odds of transition to psychosis (OR = 1.77, 95% CI [1.10, 2.83], *p* = 0.017) and lower odds of good functioning at follow-up (OR = 0.43, 95% CI [0.23, 0.81], *p* = 0.009) compared with White race.

### Relationship between ACEs and symptom remission

**Fig. 1:**
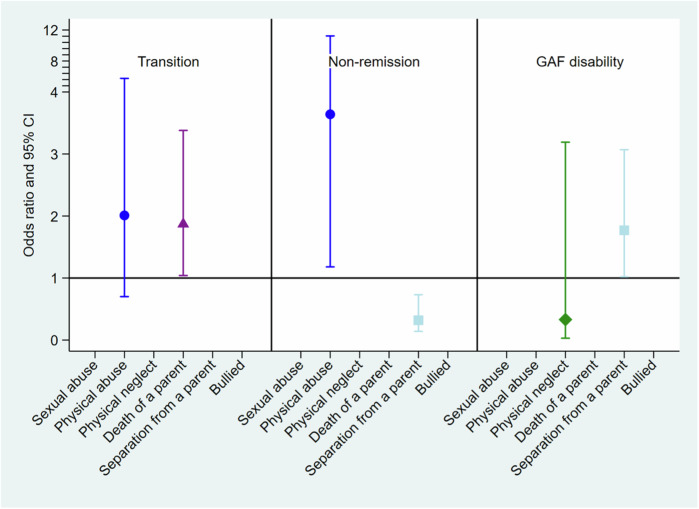
Adjusted odds ratios (adjOR) and 95% confidence intervals (CI) between clinical outcomes and ACEs.

Physical abuse was associated with a more than a threefold increase in odds of CHR symptoms non-remission (OR = 3.64, 95%CI 1.18 to 11.23, *p* = 0.025). Conversely, separation from a parent substantially increased the odds of remission (OR = 0.32, 95%CI 0.14 to 0.73, *p* = 0.011). The results are detailed in Table 2 and Fig. 1.

**Table 2:** Regression models in the different outcomes.

	Transition					Non-Remission					GAF disability				
	OR	SE	*z*	*P* > *|z*|	OR [95%, CI]	OR	SE	*z*	*P* > *|z*|	[95%, CI]	OR	SE	*z*	*P* > *|z*|	OR [95%, CI]
Sex															
-female						0.75	0.18	−1.18	0.239	[0.47, 1.21]					
Age															
Race															
-Black	1.77	0.42	2.38	**0.017**	[1.10, 2.83]	1.16	0.34	0.50	0.615	[0.65, 2.06]	0.43	0.14	−2.63	**0.009**	[0.23, 0.81]
-Mixed	0.56	0.22	−1.49	0.136	[0.26, 1.20]	0.69	0.43	−0.60	0.550	[0.20, 2.33]	0.61	0.30	−1.00	0.317	[0.23, 1.60]
-Asian	0.81	0.81	−0.21	0.837	[0.12, 5.68]	2.06	2.09	0.71	0.477	[0.28, 15.07]	1.65	1.10	0.75	0.454	[0.44, 6.10]
-other	1.26	1.34	0.22	0.830	[0.15, 10.20]	0.36	0.091	−4.03	**<0.001**	[0.22, 0.60]	0.63	0.24	−1.21	0.227	[0.30, 1.33]
IQ											1.01	0.01	1.56	0.118	[0.99, 1.03]
Sexual abuse															
Physical abuse	2.01	1.08	1.30	0.193	[0.70, 5.75]	3.64	2.09	2.25	**0.025**	[1.18, 11.23]					
Physical neglect											0.33	0.38	−0.95	0.342	[0.03, 3.19]
Death of a Parent	1.87	0.56	2.09	**0.037**	[1.04, 3.38]										
Separation from a parent						0.32	0.13	−2.69	**0.007**	[0.14, 0.73]	1.77	0.50	2.05	**0.040**	[1.02, 3.07]
Bullied															

Remission 0= yes, 1= no remission. Transition 0= no, 1= yes. GAF disability 0= low scores, 1= high scores.

The blank line indicates that this variable was not included in the final model due to lack of statistical significance.

*OR* odds ratio, *SE* standard error, *CI* confidence interval, *IQ* intelligent quotient, *CHR-T* clinical high-risk transition.

### Relationship between ACEs and functioning

Individuals who experienced separation from a parent had 1.77 times the odds of presenting with good functioning as measured with the GAF disability scale compared to individuals who had not experienced parental separation (OR = 1.77, 95% CI 1.01–3.07, *p* = 0.040). HC were finally excluded from the model because their status perfectly predicted the functioning, as all the controls presented high functioning, resulting in no variation for the model to estimate an effect (Table 2 and Fig. 1).

### Relationship between ACEs and transition to psychosis

Individuals reporting the death of a parent presented increased odds of transition to psychosis (OR = 1.87, 95% CI 1.04–3.38, *p* = 0.037), (Table 2 and Fig. 1).

## Discussion

To the authors’ knowledge, this is the first study to examine the relationship between ACEs and long-term outcomes in a large multi-centre cohort of individuals at CHR for psychosis, including participants with diverse backgrounds and ethnicities from Europe and South America. As expected, based on previous studies^56–58^, the CHR group reported significantly higher levels of several different forms of ACEs (CTQ total score, all five trauma subtypes, CECA trauma subtypes, and bullying). Our hypotheses, that within the CHR group, ACEs would be associated with adverse clinical outcomes, were partially confirmed.

Physical abuse was associated with a reduced likelihood of remission from the CHR state. Several studies have related the experience of ACEs to higher chances of suffering a mental health condition in adulthood^59–61^. Physical abuse, particularly in the formative years, can have profound and long-lasting effects on psychological well-being and development. This type of trauma can disrupt the psychological development and the ability to regulate emotions and distress^62^. Research has shown that individuals who have experienced physical abuse may have a greater vulnerability to a range of mental health disorders^63^. The trauma stemming from the abuse may lead to changes in emotion regulation, stress response, and impulse control^64^. For example, it can alter the hypothalamic-pituitary-adrenal (HPA) axis, leading to a heightened stress response that could exacerbate symptoms of mental health disorders^65^. Physical abuse can also erode trust in others^66,67^, potentially preventing or delaying help-seeking when distress arises and making the formation of therapeutic relationships — a crucial aspect of many mental health treatments — more challenging.

Contrary to our hypothesis, childhood separation from a parent was associated with increased probability of CHR symptom remission and with a higher level of functioning and was not associated with transition to psychosis. Importantly, while these associations are significant, these participants still met intake criteria for CHR status. Both groups - those with and without parental separation – demonstrate individual vulnerability to developing a mental health disorder. This vulnerability might also be influenced by other risks factors, including genetic predisposition, exposure to other ACEs or a combination of these factors. Nonetheless, these counter-intuitive findings might be explained by the child’s removal from a challenging or difficult family environment^68,69^. Past studies have reported greater odds of psychosis and other mental health conditions following parental separation^70–72^. However, children who have navigated the challenges of parental separation often develop a greater degree of resilience^72,73^. The process of adapting to significant life changes can foster coping skills and psychological strength, which might aid in recovery from mental health conditions. Childhood separation from their parents due to difficulties within the family could often mean being placed in more supportive environments, whether with other family members, in foster care, or through adoption, along with increased access to mental health resources. These environments and therapeutic services can provide the emotional support and stability needed for the child to thrive and for mental health symptoms to remit^74^. Parental separation changes the family dynamics and might relieve the child from certain dysfunctional roles they might have adopted, i.e. acting as a caretaker or mediator, roles that can be associated with distress and anxiety^75^. For some, the experience of separation and the subsequent challenges can lead to significant personal growth and development, while for others, separation from a parent can mainly be a source of trauma and emotional distress^72^.

CHR individuals who had experienced the death of a parent before age 11 had more than two-fold increase likelihood of later transition to psychosis. Consistent with this finding, a recent large multi-centre study that investigated the relationship between early parental death in childhood and psychosis reported significantly greater odds of psychosis with parental loss and even greater odds with the loss of both parents^76^. The experience of losing a parent before reaching adulthood is among the most traumatic events one can endure, with a significant increase in the risk of adjustment, psychotic, and personality disorders, among others^77,78^. The environment and conditions a child faces after the loss of a parent may have a greater long-term impact on their mental health than the event of death itself^76^, and these post-loss conditions can either mitigate or intensify the potential negative outcomes. On the other hand, a recent meta-analysis reported no association between CTQ subtypes and transition to psychosis^79^, with the exception of sexual abuse, which was correlated with a higher likelihood of transition. Furthermore, this meta-analysis reported high heterogeneity among the included studies and follow-up periods ranging from 6 months to 15 years, which limits the interpretation of the results. An earlier investigation of individuals drawn from the same cohort reported that emotional abuse was associated with transition to psychosis^80^. However, in the latter study, follow-up of the CHR sample had not been fully completed, with a duration of only 24 months, and only 11.9% had developed psychosis at the time of analysis. In the present study, the follow-up period was up to 5 years, and 11 participants that had previously been classed as non-transitioned had gone on to develop psychosis, increasing the transition rate to 17%. These differences are likely to account for the non-replication of this preliminary finding.

Finally, even though it was not the primary focus of our study, it is important to highlight the significant association found between Black race and poorer outcomes at follow-up, including lower functioning and higher odds of transitioning to psychosis. This finding aligns with previous literature indicating that ethnic minority and migrant groups are at greater risk of mental health difficulties, particularly psychosis, with Black individuals being at the highest risk^81^. Several explanations have been proposed, including the impact of racial harassment and discrimination experienced by minority groups^82^, the disproportionate risk of deprivation^83^, and, in some cases, the migration processes, which can be a traumatic experience itself^84^.

ACEs have been consistently associated with poor mental health, including and not limited to the development of psychosis. ACEs are also significantly more prevalent in CHR than in HC. ACEs seem to therefore be good candidate targets for preventive interventions^85,86^. Specifically, it is essential to understand the role of protective factors against ACEs in individuals at CHR. Previous studies highlight the protective potential of community engagement and mother-child relationships in mitigating negative outcomes, even after an adverse event has occurred^78^. However, it remains unclear whether these factors could also serve as protective mechanisms in preventing the development of psychotic symptoms.

Furthermore, therapeutic interventions may need to be tailored to address the specific needs of individuals who have experienced ACEs, and treatment plans may need to be more intensive and longer in duration. Given the high prevalence of ACEs in individuals at CHR; interventions targeting this population should always aim to be trauma-informed. Additionally, for those with comorbid PTSD, trauma-focused interventions should be offered. These include Eye Movement Desensitisation Reprocessing, which is being implemented within some early intervention for psychosis services with some positive results^87–89^, as well as prolonged exposure treatments, which have demonstrated equal or superior efficacy in populations within the psychosis spectrum^90,91^. Interventions based on cognitive processing within the framework of cognitive-behavioural therapy have also demonstrated a robust effect on trauma-related symptoms and cognitions in psychotic disorders^92,93^.

Finally, early preventive interventions, including school-based interventions able to reach most children and adolescents regardless of their ethnic and financial background, intensive support and interventions within the family of origin, temporary or prolong foster care in those cases where it is not possible to successfully support the original family, might be appropriate^72,94–96^.

### Limitations

Although information on ACEs was collected using well-established instruments (CTQ, CECA, and the Retrospective Bullying Questionnaire) in adolescents and young adults who had not yet developed psychosis, the assessments were still retrospective and can therefore be affected by factors such as recollection, repression or reporting biases^13,97^. Nevertheless, previous findings support the predictive validity of retrospective measures of ACEs on clinical outcomes^98^ and indicate that retrospective assessments are more likely to underestimate rather than overestimate the prevalence of ACEs^97^. Another potential limitation is the use of the GAF to assess functioning, as it blends clinical symptoms, social functioning, and academic/role functioning into a single score. This approach may obscure nuanced differences in functional profiles, particularly in CHR populations, where functional decline is less pronounced than in progressed psychosis and profiles are often imbalanced (e.g., intact social functioning alongside declining academic performance). Missing data at follow up, especially in relation to the variables of symptom remission and functioning, as well as the low number of events in some of the included ACEs might have reduced the sample’s statistical power. A larger sample would therefore be appropriate, in particular, to replicate our findings that childhood separation from a parent is associated with increased probability of CHR symptom remission, and with a higher level of functioning. The statistical analysis employs a stepwise regression model, which can introduce biases in variable selection and increase the risk of overfitting. To minimize this risk, covariates for the analyses were chosen based on factors that have shown a strong association with the outcomes in prior literature. Nevertheless, it remains essential to replicate these findings in independent samples to confirm their robustness. Finally, ACEs, their timing, their influence and relationship with one another, are incredibly complex and have an impact on biological, psychological and social pathways^99,100^. A much larger multi-modal dataset with detailed premorbid and longitudinal data would allow the in-depth analysis of the effects of ACEs on the bio-psycho-social systems.

## Conclusions

Our findings indicate that some ACEs in the CHR population are associated with adverse clinical outcomes. Although the relationship between ACEs and psychosis is complex, addressing ACEs within early intervention services, for example offering trauma-focused interventions, could be beneficial. Additionally, preventive strategies targeting early childhood and adolescence within the school environment might have the potential to mitigate or modify the effects of ACEs on long-term outcomes. Further research is needed to confirm this association and to refine intervention approaches.

## Data Availability

The data that support the findings of this study are not publicly available due to restrictions from the ethical approvals obtained, which do not include provisions for data sharing. Therefore, the data cannot be shared.
